# Deployment of Distributed Applications in Wireless Sensor Networks

**DOI:** 10.3390/s110807395

**Published:** 2011-07-25

**Authors:** Virginia Pilloni, Luigi Atzori

**Affiliations:** Department of Electric and Electronic Engineering, University of Cagliari, Cagliari 09123, Italy; E-Mail: virginia.pilloni@diee.unica.it

**Keywords:** Wireless Sensor Networks, energy consumption, network lifetime

## Abstract

The increase in computation and sensing capabilities as well as in battery duration of commercially available Wireless Sensors Network (WSN) nodes are making the paradigm of an horizontal ambient intelligence infrastructure feasible. Accordingly, the sensing, computing and communicating infrastructure is set with a programmable middleware that allows for quickly deploying different applications running on top of it so as to follow the changing ambient needs. In this scenario, we face the problem of setting up the desired application in complex scenarios with hundreds of nodes, which consists of identifying which actions should be performed by each of the nodes so as to satisfy the ambient needs while minimizing the application impact on the infrastructure battery lifetime. Accordingly, we approach the problem by considering every possible decomposition of the application’s sensing and computing operations into tasks to be assigned to each infrastructure component. The contribution of energy consumption due to the performance of each task is then considered to compute a cost function, allowing us to evaluate the viability of each deployment solution. Simulation results show that our framework results in considerable energy conservation with respect to sink-oriented or cluster-oriented deployment approaches, particularly for networks with high node densities, non-uniform energy consumption and initial energy, and complex actions.

## 1. Introduction

**Figure 1.**
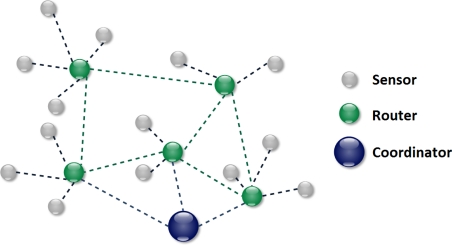
Structure of a WSN.

Over the past decade, Wireless Sensor Networks (WSN) have consistently evolved into more complex distributed monitoring and control systems. In the beginning, the only goal of a WSN was to monitor a given environment. As depicted in Figure 1, sensors gathered the required information mostly according to a fixed temporal schedule and sent it to the routers, whose primary functions were to both perform the sensing and conveniently transmit data received from sensors. Finally, the Coordinator would collect the data transmitted by the router. The Coordinator has much higher processing and memory capacity than the other nodes in the network. It initializes and manages the WSN: it is responsible for routing and storing the routing tables as well as information about the network and security keys. The Coordinator usually interfaces with a server or a computer. In this way, data from sensors could be processed and stored. The Coordinator possessed all the intelligence of the network. Given the cost of more advanced devices, all other nodes only had basic processing and memory capacity [1].

A WSN is characterized by the presence of one or more sinks. A sink is a node which gathers and controls data collected by different sensor nodes. In every network there is at least one sink present, corresponding to the Coordinator.

WSNs are now becoming more and more complex. According to the information gathered by the sensors, the network is capable of making decisions and acting upon them. Indeed, they are expected to be one of the pillars of the Internet of Things (IoT) paradigm [2], which foster the introduction of key applications, including but not limited to domotics, assisted living, e-health, enhanced learning automation and industrial manufacturing logistics, business/process management, and intelligent transportation of people and goods. Reduction in the cost of the devices has increased the nodes’ capacity, thus they can perform some processing before sending the data to a sink. They can aggregate data coming from different sensors, perform temporal and spatial averaging as well as data filtering so as to reduce the burden of transmitting large amounts of data to the Coordinator and increase the network lifetime. Indeed, devices in a WSN are typically powered by batteries that can be difficult to replace, such as in the case of subterranean or underwater nodes.

These considerations contribute to the vision of an horizontal ambient intelligence infrastructure wherein the sensing, computing and communicating infrastructure is set with a programmable middleware that allows for quickly deploying different applications running on top of it so as to follow the changing ambient needs, for example: monitoring a given geographical area and alerting when something is happening therein; activating the heating system when the ambient is getting cold; tracking the processing chain in industrial plants to prevent hazardous scenarios. In this case, we focus on the need for a logic that, starting from the desired application, can be set up in complex scenarios with hundreds of nodes, evaluate the possible deployment solutions and decide which action should be performed by each of the nodes so as to satisfy the ambient needs while minimizing the application’s impact on the infrastructure’s battery lifetime. Accordingly, we approach the problem by considering every possible decomposition of the application’s sensing and computing operations into tasks to be assigned to each infrastructure component. The contribution of energy consumption due to the performance of each task is then considered to compute a cost function allowing us to evaluate the viability of each deployment solution.

This paper is organized as follows. The second Section describes how other authors have dealt with the problem of maximizing of the network lifetime, and the model of energy consumption considered in this work. The third Section introduces the problem and how we have approached it. The following Section defines the algorithm used by the framework to maximize the network lifetime. In the fifth Section, some simulation results on the effectiveness of the framework are presented. The last Section draws final conclusions. Finally, in the Appendix we provide the list of symbols used to make it easier to follow the description of the proposed algorithm.

## 2. Background

### 2.1. Past Studies

Due to their scarce resources, minimization of energy consumption has been a key challenge for Wireless Sensor Networks. There are a great number of works that have focused on the maximization of the network lifetime, each taking into account a different approach to achieve it.

Routing is probably the most immediate issue addressed in order to accomplish this goal. A convenient choice of paths to route data may result in significant energy conservation. In [3], an energy-efficient metric for finding routes was proposed. In [4], nodes energy reserves were taken into account to route the traffic so that the nodes’ drain-out is maximized. Other routing techniques are shown in many other studies, such as [5] and [6].

Some studies build on the assumption that transmission energy consumption is related to the square of the distance between two communicating nodes. Therefore, it might be more energy efficient to send data over many short hops rather than fewer long hops. This issue was handled in [7] and, later, in [8]. This approach intends to maximize the network lifetime by minimizing overall energy consumption. However, it does not resolve the problem of unbalanced energy consumption among the nodes. This uneven energy dissipation may lead to an early death of some nodes, indeed resulting in a reduction of the network lifetime rather than an increase.

In order for some nodes not to die much earlier than others, energy consumption in the network should be as balanced as possible. Relay nodes might be used for this purpose, as shown in [9] and [10]. Energy load distribution can also be achieved by conveniently deploying the network nodes, as in [11]. In [12], the nodes are spaced non-uniformly as a function of their distance. Taking into account that nodes near the base station feel the effects of higher traffic more than other nodes, spacing is adjusted in such a way that nodes with higher traffic have a shorter hop distance than nodes with less traffic.

None of the studies mentioned above considers the possibility of processing the data in the nodes of the path to the destination. Because most of the energy spent in a Wireless Sensor Network depends on the amount of data that is transmitted over the network, reducing the amount of data may result in a reduction of the transmission energy consumption. This principle has been only partially adopted by LEACH [13], where sensors serve as Cluster Heads aggregating the data and, indeed, decreasing the number of bytes sent over the network. Energy consumption balancing is guaranteed by a random rotation of the role of Cluster Head.

In [14], Cluster Heads for a mobile WSN are chosen according to a selection algorithm which takes into account characteristics of the nodes such as power energy and transmission rate. The clustering algorithm presented is periodically re-run to possibly find new nodes which might improve the network lifetime.

Given the computational capacity of modern sensors, a step forward could be taken not just by aggregating data, but by processing them before they arrive at their destination whenever possible and on the basis of the network topology and power resource detection. In this paper, a framework that determines which of the nodes should process the data in order to maximize the network lifetime is presented. To the best of the authors’ knowledge, no similar frameworks for WSN have been proposed before. An example of an overlaying framework that handles an architecture for an integration of the IoT in enterprise services might be found in [15]. However, this framework is not conceived to minimize the energy consumption, unlike the framework hereafter described.

### 2.2. Energy Consumption

Energy consumption in Wireless Sensor Networks is determined most of all by transmission and reception. As mentioned in [16]

(1) $$\{\begin{array}{l} P_{T}(\delta )=P_{T0}+P_{A}(\delta )=P_{T0}+P_{Tx}(\delta )/\eta \\ P_{R}=P_{R0} \end{array}$$

where *P_T_* and *P_R_* are radio frequency power consumptions for transmitting and receiving respectively; *P_A_* is the power consumption of the Power Amplifier (PA); *δ* is the distance between the transmitter and the receiver; *P_T_*_0_ and *P_R_*_0_ are the components of power consumption of the transmitting and receiving circuitry respectively; P*_Tx_* is the output power at the antenna which, for reliable transmissions, depends on the distance *δ; η* is the drain efficiency of the PA.

Considering a channel in which the path loss component is predominant, and thus secondary effects such as multipath and Doppler can be neglected, the received power *P_Rx_* can be expressed as

(2) $$P_{\mathrm{Rx}}=\frac{P_{\mathrm{Tx}}}{A\times \delta^{\alpha }}$$

where *A* is a parameter determined by the characteristics of the antennas (such as gain and efficiency) and *α* denotes the path-loss exponent, which is about 2 for free space. This kind of modelling is typical of free space propagation. Of course, the model might be extended to account for other fading effects.

From Equations (1) and (2)

$$P_{T}(\delta )=P_{T0}+\frac{P_{\mathrm{Rx}}\times A\times \delta^{\alpha }}{\eta }$$

Considering *ε* = *P_Rx_min__* × *A*, where *P_Rx_min__* is the minimum reception power for a reliable communication

$$P_{T}(\delta )=P_{T0}+\frac{\varepsilon \times \delta^{\alpha }}{\eta }$$

This implies that the total power consumption for communicating between a transmitting node *A* and a receiving node *B* of a WSN could be written as

$$P_{\mathrm{AB}}=P_{R0_{B}}+P_{T0_{A}}+\frac{\varepsilon_{\mathrm{AB}}\times \delta_{\mathrm{AB}}^{\alpha }}{\eta_{A}}$$

Therefore, the energy consumption of the network to transmit a packet of *k* bits from *A* to *B* with a constant data rate *R* is

(3) $$\begin{array}{ll} e^{\mathrm{tx}}(k,P_{T0_{A}},\eta_{A},P_{R0_{B}},\varepsilon_{\mathrm{AB}},\delta_{\mathrm{AB}}) & =\frac{P_{\mathrm{AB}}\times k}{R}\\ & =\frac{k}{R}\left(P_{R0_{B}}+P_{T0_{A}}+\frac{\varepsilon_{\mathrm{AB}}\times \delta_{\mathrm{AB}}^{\alpha }}{\eta_{A}}\right)\\ & =k\times (e_{R_{B}}+e_{T_{A}}(\delta )) \end{array}$$

where *e_T_A__* is the energy to send one bit over a distance *δ_AB_* and *e_R_B__* is the energy to receive one bit.

The model described does not take into account mechanisms such as sleep schedule and route discovery, which may produce overhead. Thus it could thus be necessary to consider not just the single packet transmission, but also the energy consumption due to the overhead.

Besides transmission and reception, the other two main causes of energy consumption are due to the sensing activity and to the processing. The sensing energy consumption *e^sens^* is determined by the specific characteristics of the sensor, which is given by the used hardware.

The processing energy consumption *e^proc^*(*task, data^in^*) is proportional to the complexity of the *task*—that is, the number of instructions needed to complete the *task*—and to the ingress data *data^in^*—the higher the number of samples involved in the processing, the higher the energy consumption. This function *e^proc^*(.) can be determined on the basis of the device datasheet used. Calling *M^task^* the number of instructions for the *task, smp^in^* the number of samples to be processed and *e^instr^* the average energy consumption per instruction executed

(4) $$e^{\mathrm{proc}}(\mathrm{task},{\mathrm{data}}^{\mathrm{in}})={\mathrm{smp}}^{\mathrm{in}}\times M^{\mathrm{task}}\times e^{\mathrm{instr}}$$

A summary of the notation used throughout this paper is provided at the beginning of this document for quick reference.

## 3. Problem Formulation

**Figure 2.**
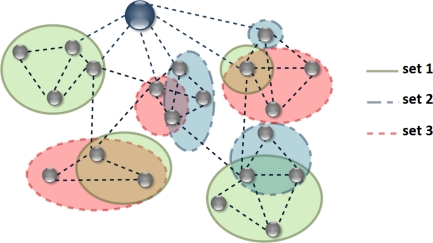
Example of a WSN. Nodes belonging to set 1 might perform task *f*_1_; nodes in set 2 might perform task *f*_2_; nodes in set 3 might perform task *f*_3_.

The goal of a WSN is to accomplish a given number of operations mostly based on some measurements performed on the relevant environment. In our scenario not all the nodes have the same capacities, as represented in Figure 2, where three sets of possible tasks have been considered, which could be: data processing, temperature measurement and video monitoring. Given the status of the network in terms of node capacities, topology, and energy distribution, the problem addressed is to assign to each node the tasks that, combined together, contribute to the target network operations while minimizing the application impact on the infrastructure battery lifetime.

In our modelling, the number of nodes *X* = {*x*_1_, ..., *x_i_*, ..., *x_N_*} in the WSN is denoted with *N*, where the node *x_i_* can be a sensing node, a router or an actuator (or node with a combination of these roles). The node *x_N_* refers to the sink (we assume there is only one sink in the network). The network can be described by:

- the *N* × *N* adjacency matrix **A** = (*a_ij_*): an element *a_ij_* of **A** is equal to 1 when a link connects node *i* to node *j* and the sink is closer to *j* than to *i*;
- the distance matrix **Δ** = (*δ_ij_*), which contains the pairwise distances (in meters) between adjacent nodes. If *δ_ij_* = ∞, nodes *i* and *j* are not adjacent;
- the matrix **E** = (*ε_ij_*), with the parameters *ε_ij_* introduced in Section 2.2, calculated for each combination of adjacent nodes *i* and *j*, where *i* reaches the sink though *j*. If *ε_ij_* = ∞, nodes *i* and *j* are not adjacent;
- the set of characteristic parameters *V_i_* *=* {*P_R_*_0_*_i_*, *P_T_*_0_*_i_*, *η_i_*} of every node *x_i_*, which are useful to compute the transmission energy consumption as defined in Equation (3) in Section 2.2;
- the set *F* = {*f*_0_, ..., *f_w_*, ..., *f_W_*} of tasks, which encompasses all the tasks that can be performed by any node in the network. For instance, 0 might correspond to “temperature sensing in the area 1”, 1 to the “temperature sensing in the area 2”, 2 to the “pressure sensing in the area 3”, 3 to the “spatial averaging” (which means performing the average of the sensed data arriving from different geographical areas), 4 to the ‘temporal averaging” (which means performing the average of sample values sensed by the same sensor at different instants of time), 5 to the “only transmission”, 6 to “no actions”. Each of the tasks in an *F* set entails a transmission of data, with the exception, of course, of “no actions”;
- the set *D_i_* = {*d_i_*_1_, ..., *d_im_*, ..., *d_il_i__*}, with *D_i_* ⊆ *F*, where the elements of *D_i_* are the tasks that the node *x_i_* is able to perform.

We assume that a given operation *O* has to be deployed in the network, which can be decomposed into a sequence of distributed tasks. This could represent diverse operation, including: computing the average of the temperature in certain geographical areas, measuring the light intensity in a room, video-surveillance of a specific geographical area, or a combination of these. In the following, we rely on a specific reference application, which we name *spatial and temporal monitoring*: a spatial and temporal mean operation over an hour is performed on the temperature values sensed by the sensors every 10 min by the sensors from 3 different locations; the average values are stored in the sink. We use this example to better explain our modelling.

Three significant parameters can be associated with the operation to be deployed:

- the total cost value *E^tot^*, which takes into account the energy consumption;
- the sequence *C* = {*c*_1_, ..., *c_l_*, ..., *c_L_*} of the sub-operations that must be executed by the nodes to perform the operation *O*. If, for instance, *O* is our reference example of *spatial and temporal monitoring*, some of the sub-operations in *C* will certainly be “temporal averaging” and “spatial averaging”. Each sub-operation is equivalent to one of the *f_w_* tasks defined above: *C* ⊆ *F.* The terms sub-operation and task are used interchangeably, but we refer to *c_l_* as a sub-operation to emphasise its link with its respective operation *O*. Sub-operations are listed in *C* in order of priority: if a node has to execute both *c*_1_ and *c*_2_, the former must be executed before the latter. With reference to our *spatial and temporal monitoring* example, *c*_1_, *c*_2_ and *c*_3_ are the sensing sub-operations: *c*_1_ is “temperature sensing in area 1”, *c*_2_ is “temperature sensing in area 2” and *c*_3_ is “temperature sensing in area 3”; because every sensor only belongs to one area, the order of the sensing sub-operations is irrelevant. Rather, it is important that data is gathered before any other sub-operation is performed on it; thus, the sensing sub-operations are the first elements in *C*. If a node has to calculate both the temporal and spatial mean of the received values, it has to first work out the temporal mean on the data received from each path, and then it has to perform the spatial mean on it. Therefore, *c*_4_ is “temporal mean” and *c*_5_ is “spatial mean”. Finally, *c*_6_ is “only transmission” and *c*_7_ is “no actions”. All the sub-operations for the example are summarized in Table 1;
- the set *S* = {*s*_1_, ..., *s_i_*, ..., *s_N_*}, where *s_i_* is the status of node *i* with respect to operation *O*. The value *s_i_* defines which sub-operation *c_l_* the node *i* is performing. Of course, the status of the node *x_i_* has to be chosen among the set of tasks *D_i_* that the node is able to perform. If the node is not involved in the operation *O*, its status corresponds to “no actions”. For this reason, “no actions” is necessarily included in set *F*. The following must always be verified:
  – if a node *x_j_* receives some data from a node *x_i_*, which means that *s_i_* cannot be “no actions”, node *x_j_* must at least transmit the data
    (5) $$s_{i}\neq "\text{no actions}"\;\vee a_{\mathrm{ij}}=1\Rightarrow s_{j}\neq "\text{no actions}"$$
  – if a node *x_j_* does not receive any data, which means that the status of all the nodes connected to it is “no actions”, *s_j_* must also be set to “no actions”
    (6) $$s_{i}\equiv "\text{no actions}"\;\forall i:a_{\mathrm{ij}}=1\Rightarrow s_{j}="\text{no actions}"$$

Thanks to the greater processing power and storage capacity of modern sensors, contrary to the past, the same operation *O* can be performed in several different ways: gathered data can be immediately sent to a sink or it can be processed before being transmitted. In the case of the latter, the number of bits to be sent would be smaller, and therefore the transmission energy consumption would be lower as well; however, processing energy consumption could be higher in this second case. Quantifying the energy consumption in both cases, it could be possible to establish which one determines a reduction of battery consumption in the sensors, incrementing the network lifetime.

The framework described further takes a high-level code as input, evaluates which combination of statuses *S* = {*s*_1_, ..., *s_i_*, ..., *s_N_*} permits the operation *O* to be performed with the lowest possible energy consumption *E^tot^*, and finally elaborates and assigns among the nodes the most appropriate tasks to be performed. Hence, it is evident that the cost function *E^tot^* will vary depending on the status of each node, that is, how the operation *O* is performed. The problem addressed is then defined as the set of statuses *S* that minimizes the impact of the application on the network lifetime. In the following, we elaborate the considered scenario by defining further constraints that solve the problem.

## 4. Deployment of Distributed Applications

In the following, we present the proposed solution towards a distributed application deployment in WSN. The following Subsections present: the constraints on the traffic generated by the distributed applications; the concept of virtual nodes, which are duplicates of real nodes that are introduced to deal with nodes that perform more than a single action in a deployment solution; the cost functions built on the basis of the energy consumption formulae; the network lifetime maximization procedure; a summary of the proposed framework. Note that the modelling that we propose in this work and that we present in this Section is aimed at evaluating all the possible solutions of application deployment in terms of data transmission and processing. The parameters, constraints and cost functions are introduced for the sole aim of evaluating the viability of the solution.

### 4.1. Constraints on Traffic Flows

In our scenario we assume that the sources of traffic in the network (the sensors) generate samples of *k* bits at a certain frequency *f*. The processing in the network is performed on this type of traffic flow coming from different nodes. The generic node *x_i_* receives the traffic $T_{i}^{\mathrm{in}}$ over which it performs the sub-operation corresponding to its assigned status *s_i_*. The effect of this sub-operation is the generation of the output traffic $T_{i}^{\mathrm{out}}$, which is computed by function *p* as follows

(7) $$T_{i}^{\mathrm{out}}=p\left(T_{i}^{\mathrm{in}},s_{i}\right)$$

The output traffic is then sent to the next node towards the sink.

The data generated by *p* in the node *x_i_* is modelled by the *H*-dimensional vector $T_{i}^{\mathrm{out}}=\left(t_{i1}^{\mathrm{out}},\ldots ,t_{\mathrm{ih}}^{\mathrm{out}},\ldots ,t_{\mathrm{iH}}^{\mathrm{out}}\right)$, where each element $t_{\mathrm{ih}}^{\mathrm{out}}=\{k_{\mathrm{ih}}^{\mathrm{out}},f_{\mathrm{ih}}^{\mathrm{out}}\}$ corresponds to a traffic flow where each sample of $k_{\mathrm{ih}}^{\mathrm{out}}$ bits is transmitted at the frequency $f_{\mathrm{ih}}^{\mathrm{out}}$. Each sample described by $t_{\mathrm{ih}}^{\mathrm{out}}$ results from a spatial processing or a sensing. The data $T_{i}^{\mathrm{out}}$ is then sent to the following node *x_j_*, according to adjacency matrix **A**.

The node *x_j_* receives data from all adjacent nodes that reach the sink through *x_j_*, with the exception of the nodes with a “no actions” status

(8) $$T_{j}^{\mathrm{in}}=\overset{N}{\underset{i=1}{\cup }}T_{j}^{\mathrm{out}}\times a_{\mathrm{ij}}\times z_{i},\;\;\;\;\;\;\;\;\;\;\;\;\text{with}\;z_{i}=\{\begin{array}{ll} 0 & s_{i}\equiv "\text{no actions}"\\ 1 & \text{otherwise} \end{array}$$

As defined by Equation (7), the data $T_{j}^{\mathrm{in}}$ received by the node *x_j_* is processed, according to the status of *x_j_*:

- if *s_j_* is a sensing status, *p* does not take any $T_{j}^{\mathrm{in}}$ as input and the output is defined by the specific sensing operation;
- if *s_j_* is an “only transmission” status, the output of *p* is exactly equal to $T_{j}^{\mathrm{in}}$;
- if *s_j_* is a “no action” status, *p* returns a $T_{j}^{\mathrm{out}}$ with all fields set to 0 as output. This case is included for completeness, but it is not supposed to happen because of Equation (5);
- if *s_j_* is a processing status, $T_{j}^{\mathrm{out}}$ can be the most diverse depending on the specific processing objectives, which are coded in *s_j_* and that control the specific function *p*. In the following we analyze certain cases.

**Figure 3.**
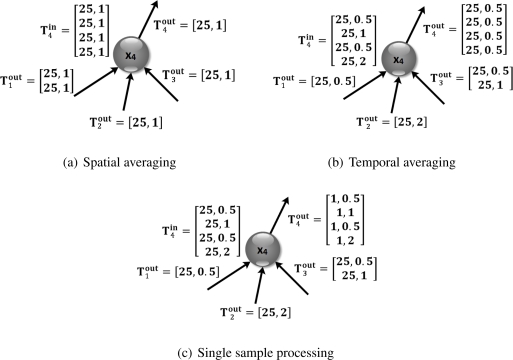
Examples of data processing in node 4 that receives input traffic from nodes 1–3. In these sketches we show the input and output traffic for: spatial averaging (**a**); temporal averaging (**b**); and single sample processing (**c**).

Referring to the *spatial and temporal monitoring* example, processing can be a spatial averaging, a temporal averaging, or a combination of both. In a spatial processing, the samples coming from different paths are processed together, as shown in an example in Figure 3(a). Here, four flows of 25 bits per second are received by node 4, which are then averaged to produce a single flow of 25 bits per second. Accordingly, the resulting $T_{j}^{\mathrm{out}}$ is made of only one element $t_{j1}^{\mathrm{out}}=\left\{k_{j1}^{\mathrm{out}},f_{j1}^{\mathrm{out}}\right\}$, where the number of bits per sample $k_{j1}^{\mathrm{out}}$ and the frequency $f_{j1}^{\mathrm{out}}$ are equal to those of each input flows. It must be noted that, in general, $k_{j1}^{\mathrm{out}}$ is not necessarily equal to the number of bits of each input flow, but it may be different according to the processing output.

Differently, the temporal averaging is performed on every traffic flow in $T_{j}^{\mathrm{in}}$. The resulting $T_{j}^{\mathrm{out}}$ contains the same number of traffic flows as in $T_{j}^{\mathrm{in}}$, where every element $t_{\mathrm{jh}}^{\mathrm{out}}$ is characterized by the same number of bits per sample $k_{\mathrm{jh}}^{\mathrm{out}}$ and the same frequency $f_{\mathrm{jh}}^{\mathrm{out}}$ corresponding to the averaging frequency associated to the node status *s_j_*. Indeed, we may have different status codes associated to the temporal averaging, each one distinguished by a different processing frequency. Figure 3(b) shows an example for this kind of processing, where, in this case, we assume the node status corresponds to temporal averaging with frequency 0.5 Hz.

Other processing tasks can be performed on every single sample of each received traffic flow without involving other samples. This is the case, for instance, where one must evaluate whether the received values exceed a given threshold or not, consequently transmitting a boolean output value. The only thing that changes in the output traffic flows is the number of bits per sample; therefore, $T_{j}^{\mathrm{out}}$ contains the same number of traffic flows as in $T_{j}^{\mathrm{in}}$ at the same frequency $f_{\mathrm{jh}}^{\mathrm{out}}$, but with different bits per sample $k_{\mathrm{jh}}^{\mathrm{out}}$. Figure 3(c) shows the traffic flows for the described processing.

There are many other processing tasks that can be performed in a given network and they are coded in *F* as described in the previous Section. For each one of these, an operator *p*(*x*, *y*) is defined. Note that for our objective, this operator is needed to figure out the traffic flows that will be traversing the network for each deployment scenario.

### 4.2. Virtual Nodes

It is possible that a single node has to perform more than one task. For instance, if operation *O* is a temporal and spatial average of the temperature values measured in different geographical areas, it may happen that a single node has to compute both spatial and temporal average values on the received data. To take into account this type of scenario, we rely on the concept of virtual nodes. These are copies of real nodes, each one able to perform only a specific sub-operation and sending the resulting data at zero-energy cost to the next virtual node (except the last one that sends the data to the next node). We define the set of sub-operations that a single node *x_i_* can perform consecutively for the implementation of the operation *O*

(9) $$G_{i}=D_{i}\cap C\backslash L$$

where *L* is the set of sub-operations that the node cannot execute with the others, which are “only transmission” and “no actions”. These two are kept outside the set *G_i_* because it cannot happen that a sequence of sensing/processing tasks are followed by any of the tasks in *L*. If |*G_i_*| > 1, the node *x_i_* is divided into |*G_i_*| virtual nodes. Each virtual node is created so as to be able to execute only one of the sub-operations in *G_i_*, to which the sub-operations in *L* are added. Each virtual node can then be assigned to perform only one of these three sub-operations (the one taken from *G_i_* plus the two in *L*). Hence, the new network will have a number *N^vir^* of total nodes $x_{v}^{\mathrm{vir}}$

$$N^{\mathrm{vir}}=\sum_{i=1}^{N}\Gamma_{i},\;\;\;\;\;\;\text{with}\;\Gamma_{i}=\{\begin{array}{ll} \left|G_{i}\right| & \text{if}\left|G_{i}\right|\;>0\\ 1 & \text{otherwise} \end{array}$$

**Figure 4.**
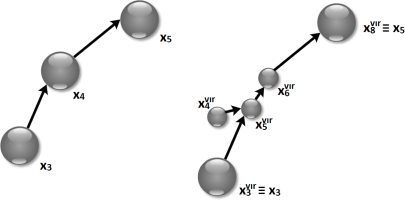
Example of virtual nodes substituting node *x*_4_. Node *x*_4_ can perform a sensing action.

The set of possible sub-operations for $x_{v}^{\mathrm{vir}}$ is $D_{v}^{\mathrm{vir}}$. Figure 4 draws an example of sequence of virtual nodes for node *x*_4_, which is substituted by nodes $x_{4}^{\mathrm{vir}}$, $x_{5}^{\mathrm{vir}}$ and $x_{6}^{\mathrm{vir}}$. Let us refer to the *spatial and temporal monitoring* example and the associated set of sub-operations *C* listed in Table 1. Additionally, let us assume that {*c*_1_, *c*_4_, *c*_5_, *c*_6_, *c*_7_} ⊂ *D*_4_, that is: the node *x*_4_ can sense the area 1, perform a temporal averaging, a spatial averaging, only transmit the received data and perform no actions (these last two tasks are always included). Because *L* = {*c*_6_, *c*_7_}

(10) $$\begin{array}{l} D_{4}^{\mathrm{vir}}=\{c_{1},c_{6},c_{7}\}\\ D_{5}^{\mathrm{vir}}=\{c_{4},c_{6},c_{7}\}\\ D_{6}^{\mathrm{vir}}=\{c_{5},c_{6},c_{7}\} \end{array}$$

A new adjacency matrix **A***^vir^* is defined to incorporate additional virtual nodes. Such matrix is built simply by substituting the real node with the sequence of virtual nodes, so that the first virtual node is connected to the nodes from which *x_i_* received the data, while the last virtual node is connected to the node to which *x_i_* sent the data. The other nodes are connected in sequence. An exception happens if the real node can also perform some sensing functions. In this case, the corresponding virtual node is kept outside this sequence and it merely sends the data to the subsequent virtual node. This rule has been introduced because the sensing operation does not need any data from other nodes. This scenario is what happens in the example shown in Figure 4, where the temperature sensing function can be performed by node $x_{4}^{\mathrm{vir}}$.

As defined in Section 3, sub-operations are in priority order. For this reason, virtual nodes have to be placed such that the first performs the sub-operation with the highest priority, and the last executes the sub-operation with the lowest priority and sends the data to the rest of the network. With reference to Figure 4, the highest priority is given to the sensing action, because whether there are any data to be sensed, the information should be gathered before processing it. For this reason, sub-operation *c*_1_ is included in $D_{4}^{\mathrm{vir}}$. If data received by node $x_{5}^{\mathrm{vir}}$ has to be processed, temporal averaging should be performed before spatial averaging. Thus, priority of the former must be higher than the latter, and this is the reason why temporal averaging *c*_4_ is an element of set $D_{5}^{\mathrm{vir}}$, while spatial averaging *c*_5_ is an element of set $D_{6}^{\mathrm{vir}}$. The adjacency matrix **A***^vir^* has to be defined accordingly.

A set of characteristic parameters $V_{i}^{\mathrm{vir}}=\left\{P_{R0i}^{\mathrm{vir}},P_{T0i}^{\mathrm{vir}},\eta_{i}^{\mathrm{vir}}\right\}$ has to be associated with each node. Taking into account the virtual nodes substituting node *x_i_*, the transmission cost from a virtual node to the other must be null. Therefore, only the last virtual node, that has to transmit the data to the network, has the same characteristic parameters $V_{i}^{\mathrm{vir}}$ as *x_i_*: the virtual nodes before it must have the parameters $P_{R0i}^{\mathrm{vir}}$ and $P_{T0i}^{\mathrm{vir}}$ set to 0.

New matrices **Δ***^vir^* and **E***^vir^* are defined, where the values of their elements for adjacent virtual nodes from the same original node are null. From now on, we will be referring only to a network with virtual nodes; however, to make the presentation clearer, we will skip subscript “*vir*”, as it is unnecessary.

### 4.3. Cost Functions

The objective of the proposed algorithm is to evaluate the viability of each deployment solution on the basis of a cost function that is connected to energy consumption. Quite often in similar scenarios, past studies have proposed the evaluation of the network lifetime and have aimed at maximizing it. Since in our framework we assume the network may be employed to perform more than one operation simultaneously, there is no sense in computing the network lifetime since it is affected by other applications which are not considered in the same analysis. For this reason we try to minimize the energy consumption for the operation under analysis, allowing the network administrator to also include a parameter that takes into account the current node battery energy level, as it is shown in the following.

We consider three cost functions: one for the sensing, one for the processing and one for the transmission. The sensing cost function for node *x_i_* is expressed as

(11) $$E_{i}^{\mathrm{sens}}=f_{i}^{\mathrm{out}}\times \gamma_{i}\times e_{i}^{\mathrm{sens}}\times y_{i},\;\;\;\;\;\;\text{with}\;y_{i}=\{\begin{array}{ll} 1 & \text{if}\;s_{i}\equiv \text{sensing code}\\ 0 & \text{otherwise} \end{array}$$

with $e_{i}^{\mathrm{sens}}$ representing the sensing energy consumption as defined in Section 2.2. Recall that $f_{i}^{\mathrm{out}}$ is the node output traffic frequency, which also represents the sensing frequency. The parameter *γ_i_* is a coefficient in inverse proportion to the residual energy of the node which can be set to drive the deployment of the application towards nodes with higher residual energy levels, as anticipated above. When performing our experiments, we have set *γ_i_* to 1 when the battery is fully loaded, while we set *γ_i_* to 5 when the battery level is lower than 20% of the total charge. From 1 to 5, *γ_i_* changes linearly.

We defined the processing cost function as follows

(12) $$E_{i}^{\mathrm{proc}}=\sum_{h=1}^{H}f_{\mathrm{ih}}^{\mathrm{out}}\times \gamma_{i}\times e_{\mathrm{ih}}^{\mathrm{proc}}(c_{s_{i}},T_{i}^{\mathrm{in}})\times v_{i}\;\;\;\;\;\;\text{with}\;v_{i}=\{\begin{array}{ll} 1 & \text{if}\;s_{i}\equiv \text{processing code}\\ 0 & \text{otherwise} \end{array}$$

where *γ_i_* is the coefficient defined above, and $e_{\mathrm{ih}}^{\mathrm{proc}}$ is the processing energy consumption defined by Equation (4), which depends on the characteristics of the node *x_i_*, the sub-operation *c_s_i__* that has to be executed, which in turn depends on the status *s_i_* of the node, and the received data $T_{i}^{\mathrm{in}}$ described in Equation (8). Because the processing cost depends on the number of processing per second performed by the same node *x_i_*, it is proportional to the frequency $f_{\mathrm{ih}}^{\mathrm{out}}$ of each of the *H* egress traffic flows, where *H* is the size of $T_{i}^{\mathrm{out}}$ as described in Section 4.1. The number of samples to calculate $e_{i}^{\mathrm{proc}}$ is defined differently for each kind of processing detected in Section 4.1. For a spatial processing, the number of processed samples is equal to the number of ingress traffic flows; for a temporal processing, the number of processed samples for each traffic flow $t_{\mathrm{ih}}^{\mathrm{out}}$ is the ratio between the frequency of arrival of the samples $f_{\mathrm{ih}}^{\mathrm{in}}$ and the processing frequency $f_{\mathrm{ih}}^{\mathrm{out}}$; by definition, for a single sample processing the number of processed samples is 1.

Both sensing and processing are followed by a transmission. Therefore, unless the node is in a “no actions” status, it has to transmit the output data. Because for Equation (5) if a node receives data it cannot be in “no actions” status, every node involved in the operation *O* has to transmit data. The related cost function is

(13) $$E_{i}^{\mathrm{tx}}=f_{i}\times \gamma_{i}\times e^{\mathrm{tx}}(T_{i}^{\mathrm{out}},V_{i},\overset{N}{\underset{j=1}{\cup }}V_{j}\times a_{\mathrm{ij}},\overset{N}{\underset{j=1}{\cup }}\varepsilon_{\mathrm{ij}},\overset{N}{\underset{j=1}{\cup }}\delta_{\mathrm{ij}})$$

where *f_i_* is the transmission frequency, *γ_i_* is the residual energy coefficient, and *e^tx^* is the transmission energy consumption defined by Equation (3), depending on: the data to be transmitted $T_{i}^{\mathrm{out}}$; the characteristic parameters *V_i_* of the node *x_i_*; the characteristic parameters *V_j_* of all the *x_j_* nodes that will receive the data from *x_i_*, which, for a connected graph, is just one; the parameter *ε_ij_* concerning nodes *x_i_* and *x_j_*; the distance *δ_ij_* between *x_i_* and *x_j_* .

Given Equations (11–13), the overall cost function for any operation *O* is

(14) $$E^{\mathrm{tot}}=\sum_{i=1}^{N}\left(E_{i}^{\mathrm{sens}}+E_{i}^{\mathrm{proc}}+E_{i}^{\mathrm{tx}}\right)$$

### 4.4. Maximization of Network Lifetime

The goal is to find the set of the statuses *S* = {*s*_1_, ..., *s_i_*, ..., *s_N_*} of the nodes that minimize the network energy cost function.

Therefore, the optimization problem becomes

(15a) $$\text{minimize}\;E^{\mathrm{tot}}=\sum_{i=1}^{N}\left(E_{i}^{\mathrm{sens}}+E_{i}^{\mathrm{proc}}+E_{i}^{\mathrm{tx}}\right)$$

(15b) $$\text{subject to}\;Q_{l}^{\text{min}}\leq \sum_{i=1}^{N}q_{\mathrm{il}}\leq Q_{l}^{\max}\;\;\;\;\;\;\;\;\text{with}\;q_{\mathrm{il}}=\{\begin{array}{ll} 1 & s_{i}\equiv c_{l}\\ 0 & \text{otherwise} \end{array}$$

(15c) $$\underset{v}{\cup }\{s_{v}\}=G_{N}\;\;\;\;\;\;\;\forall v:x_{v}\mapsto x_{N}$$

The condition in Equation (15b) is a constraint on the minimum ( $Q_{l}^{\min}$) and the maximum ( $Q_{l}^{\max}$) number of nodes that have to perform the sub-operation *c_l_*. This could be necessary, for example, when a given geographical area is monitored by a certain number of nodes, but the required information is not needed from all of them. If, for instance, the mean temperature value of an area monitored by 30 sensors is needed, we may prefer the temperature information to be gathered just by 10 of those sensors, in order to consume less energy. In this case, both $Q_{l}^{\min}$ and $Q_{l}^{\max}$ would be equal to 10, and the algorithm would choose the 10 sensors which weight less on the network lifetime, among the 30 sensors which are able to sense temperature in the required area. When this constraint is not needed for a sub-operation *l*, $Q_{l}^{\min}$ is null and $Q_{l}^{\max}$ is set to *N*.

The condition in Equation (15c) shows that the set of statuses of the virtual nodes corresponding to the original sink node *x_N_* must correspond to the set |*G_N_*|.

This implies that none of the virtual nodes corresponding to the original sink node can be in “only transmission” or “no actions” status, but they are in a processing status; therefore, if there is any data still to be processed, those virtual nodes have to process them.

The problem defined in Equation (15) is a Binary Integer Programming (BIP) problem: the unknown status of a node *x_i_* can be defined as a |*C*|-dimensional binary array, where *C* is the set of sub-operations as defined in Section 3. Because every node can only have one status, which means that it can perform only one sub-operation among those that it is able to perform, only one element of this array can be equal to 1, and it corresponds to one of the sub-operations that the relating node *x_i_* is able to perform, according to *D_i_*. The elements of the array represent the weights to the contributions in Equations (11,12) of the node to the cost function.

BIP problems are classified as NP-hard. Their exact solution is usually found using branch-and-bound algorithms. The worst case complexity of branch-and-bound algorithms is the same as the complexity of exhaustive search, which means that its complexity scales exponentially with the problem size. In the case under consideration, the problem size is dominated by the number of sub-operations |*C*| and the number of virtual nodes *N^vir^*. Therefore, the worst case complexity would be *O*(2^|^*^C^*^| ×^ *^N^vir^^* ). Nevertheless, in most cases branch-and-bound is more efficient compared to exhaustive search. Furthermore, our problem’s structure is such that only one element of the |*C*|-dimensional array representing the status of each node is nonzero. This condition allows to reduce the search space to *O*(*N^vir^*^|^*^C^*^|^). It has to be noted that commercial mathematical programming solvers such as CPLEX [17] or Xpress Optimization Suite [18] are claimed to use optimized branch-and-bound algorithms whose complexity scales linearly with the problem size.

In order to further reduce complexity, heuristic algorithms might be used as well, obtaining sub-optimal solutions which may be considered sufficient in most cases.

### 4.5. The Proposed Framework

Given a network similar to the one in Figure 2, the Coordinator, which is responsible for initiating and maintaining the network, is the device on which the deployment algorithm is performed. The proposed algorithm needs to know the exact topology of the network, that is, how the nodes are connected to each other and what the distance between any two of them is, as well as the routing table. In order to compute the cost function in Equation (14), further information is needed, such as the parameters to model the radio channel, the transmission, reception, sensing and processing energy consumption of each node, the residual energy of each node, the working frequency and the data rate.

In summary, the algorithm performs the following steps:

1. define *N x_i_*, sets *F*, *D_i_*, *V_i_* and matrices **Δ**, ***E*** and ***A***;
2. define set *O* and the corresponding set *C*;
3. define sets *G_i_*;
4. define the new network with *N^vir^* virtual nodes $x_{i}^{\mathrm{vir}}$, and new sets $D_{i}^{\mathrm{vir}}$, $V_{i}^{\mathrm{vir}}$, **Δ***^vir^*, **E***^vir^* and ***A****^vir^*;
5. given in Equation (15), solve it with a linear programming solver, in order to find set *S*.

The solving algorithm has been implemented in Mosel language, and the solution has been found using Xpress Optimization Suite. The binary array $\Sigma_{v}^{\mathrm{vir}}=\left\{\sigma_{v1}^{\mathrm{vir}},\ldots ,\sigma_{\mathrm{vl}}^{\mathrm{vir}},\ldots ,\sigma_{\mathrm{vL}}^{\mathrm{vir}}\right\}$ has been associated to each node $x_{v}^{\mathrm{vir}}$, where *L* is the cardinality of *C*, that is, the number of sub-operations which must be executed to perform operation *O*, as described in Section 3. The elements of $\Sigma_{v}^{\mathrm{vir}}$ must satisfy the following constraints:

- the element $\sigma_{\mathrm{vl}}^{\mathrm{vir}}$ can be equal to 1 if and only if $x_{v}^{\mathrm{vir}}$ is able to perform the sub-operation c*_l_* ∈ *C*, which means that $\sigma_{\mathrm{vl}}^{\mathrm{vir}}$ cannot be equal to 1 if the sub-operation *c_l_* is not an element of the set of tasks $D_{v}^{\mathrm{vir}}$ that the node $x_{v}^{\mathrm{vir}}$ is able to perform
  (16) $$c_{l}\notin D_{v}^{\mathrm{vir}}\Rightarrow \sigma_{\mathrm{vl}}^{\mathrm{vir}}\neq 1$$
- only one element in $\Sigma_{v}^{\mathrm{vir}}$ can be equal to 1
  (17) $$\sum_{l=1}^{L}\sigma_{\mathrm{vl}}=1$$

The elements of the array built this way are the weights *y_i_* and *v_i_* of the energy contributions in Equations (11,12) defined in Section 4.3.

The optimum way to perform the operation *O* at issue and spend the least amount of energy as possible will then be found. The node or the combination of nodes that are able to perform it and consume the minimum amount of energy will be chosen; then, a low level code describing which tasks each node has to perform will be developed and distributed to the appropriate nodes.

## 5. Performance Analysis

### 5.1. Test Cases and Simulations Setup

To evaluate the effectiveness of the proposed strategy, three test cases have been taken into account according to some of the most significant realistic scenarios considered in past works, such as in [13]:

1. **case1**: uniform energy consumption and uniform initial energy (UC-UE) at each node (equal characteristic parameters and battery life for every node);
2. **case2**: non-uniform energy consumption and uniform initial energy (NUC-UE) at each node (different characteristic parameters but same battery life for every node). The energy consumption of the nodes have been assigned according to a uniform distribution from 60% to 140% of the energy consumption for case 1;
3. **case3**: uniform energy consumption and non-uniform initial energy (UC-NUE) at each node (same characteristic parameters but different battery life for every node). The battery charge has been assigned randomly according to a uniform distribution from 20% to 100% of the total charge.

We monitored a rectangular-shaped outdoor environment (e.g., a vineyard, a seaport, a tourist plaza), divided into areas of 25 m^2^, where the nodes have been deployed with different densities:

- 0.2 nodes per square meter;
- 0.3 nodes per square meter;
- 0.4 nodes per square meter.

The nodes have been positioned randomly, following a uniform distribution. Each node is equipped with sensors for the measurement of temperature, humidity, PH and light exposure. The data are sent to the Coordinator, which has identification number *N.*

We have focused our analysis on the following two operations:

(OpA) calculation and storage in the sink of the (temporal and spatial) mean values of temperature, humidity, PH and light exposure over an hour, starting from the values sensed every 10 min from every area;
(OpB) aggregation of traffic coming from different areas of the network, carrying temperature, humidity, PH and light exposure values to the Coordinator for later analysis by qualified staff.

We assumed that each sensed value is represented as a double numerical value, which is 64 bits long. Note that these two operations have been selected to compare the scenarios in which the network is required to perform significant data processing (OpA) and nodes have to perform only basic processing on the data, yet can significantly reduce the amount of transmitted data by aggregating the sensed samples (OpB).

For both OpA and OpB, the first sub-operations are the sensing ones: *c*_1_ is the “temperature, humidity, PH and light exposure sensing for area 1”, *c*_2_ is the “temperature, humidity, PH and light exposure sensing for area 2”, and so on for every area. In order not to weigh down the text with alternatives, we suppose there are just 2 areas, which is not the case of the simulation scenario. Similarly to the *spatial and temporal monitoring example* in Section 3, for OpA *c*_3_ is “temporal mean”, *c*_4_ is “spatial mean”, *c*_5_ is “only transmission” and *c*_6_ is “no actions”. In addition to *c*_1_ and *c*_2_, the sub-operations for OpB are: *c*_3_ as “aggregation of samples”, *c*_4_ as “only transmission” and *c*_5_ as “no actions”. The sub-operations for the two operations are summarized in Table 2.

The nodes communicate using IEEE 802.15.4 radio interfaces on the 2.4 GHz frequency band. To keep things simple, any possible overhead has not been taken into account.

We have simulated the resulting scenarios in MatLab environment, where we have implemented the proposed algorithm together with alternative approaches as discussed in the following Subsection. The main setup parameters are listed in Table 3 ([16,19,20]).

### 5.2. Analysis of Case Studies

The optimization algorithm has been applied to each of the cases mentioned in Section 5.1. The resulting cost value has been compared with:

1. the cost value that is obtained if data are processed only by the Coordinator. This means that each traffic flow generated by the sensors is sent to the Coordinator without any processing at the intermediate nodes. We refer to this comparison with the letter “C”;
2. the data are processed (whenever needed) by every Cluster Head (node receiving flows from different sensors) found in the path to the Coordinator. We refer to this comparison with the letters “CH”;
3. the mean cost value for all possible solutions that might be detected. This is introduced to make a comparison with a possible solution where the processing of the data is performed on fixed nodes, which is expected to bring results corresponding to the median solution. We refer to this comparison with the letter “M”.

These comparisons are expressed as a percentage of the energy conservation that would result using the proposed technique with respect to the alternatives one.

**Figure 5.**
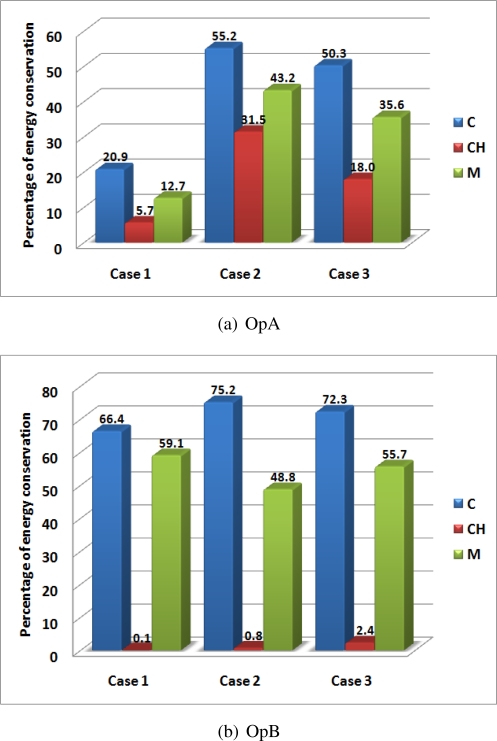
Percentage of energy conservation using the framework, for (**a**) OpA and (**b**) OpB, in cases UC-UE, NUC-UE and UC-NUE, comparisons C, CH and M, for a node density of 0.3 nodes per *m*^2^.

Figure 5 shows the results for the two operations.

**Figure 6.**
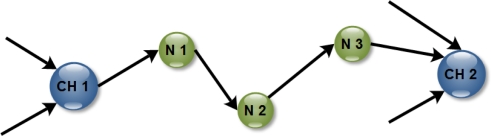
Example of a transmission from Cluster Head CH 1 to Cluster Head CH 2.

The two graphs show significant improvements of the proposed strategy with respect to the alternative ones with an average improvement of 36.3%. Limited benefits are observed in case UC-UE for both OpA and OpB. In fact, when all the nodes have the same parameter, and thus have a uniform energy consumption and the same initial energy, the choice of which node will perform the processing boils down to which Cluster Heads will do it. To illustrate the scenario, we refer to Figure 6, which depicts a Cluster Head CH 1 connected to the Cluster Head CH 2 by nodes 1, 2, and 3. Note that the Cluster Heads are just nodes that receive more than one traffic flow from different links. Because processing in CH 1 weights on the network as much as processing on node 1, node 2 or node 3, any processing of the data before arriving to CH 2 is more energy conserving. Processing the data on CH 1 ensures spending less transmission energy than processing data on nodes 1, 2 or 3. Accordingly, the CH approach allows for obtaining results similar to the ones we obtained with our approach in case UC-UE. We get slightly better results because when Cluster Heads are close to each other sometimes it is better to perform the processing only in the second Cluster Head rather than in both of them.

On the contrary, in cases NUC-UE and UC-NUE, devices’ energy consumption does not weight the same amount on the entire network. This means that the nodes chosen by the proposed algorithm to perform the processing will be those that weight less on the network, regardless of whether they are Cluster Head or not. Therefore, the detection of the lower cost solution determines the best results, in terms of energy consumption, for networks with heterogeneous parameters, which are the most common type of networks in real scenarios.

The benefits with respect to the approach CH are lower than the case of using approaches C and M. In fact, literature dictates that the use of Cluster Heads is a convenient solution, because the aggregation of frames coming from different paths leads to a reduction in network energy consumption. For this reason, when using Cluster Heads the cost is much lower, compared to sending every single frame to the Coordinator, or to the average of the other possible solutions; this determines less difference from the optimization algorithm solution, and thus a lower energy conservation. However, this approach requires every node in the network to be able to perform data processing, which is not always the case. In any case, our approach is proven to always outperform the CH approach.

It could be noted that for OpA, in which processing is more elaborate and the number of instructions for every process greater, energy conservation is higher than it is for OpB. In fact, as could be expected, the lower the energy cost necessary for the processing, the more convenient it is to process the data in every Cluster Head encountered.

**Figure 7.**
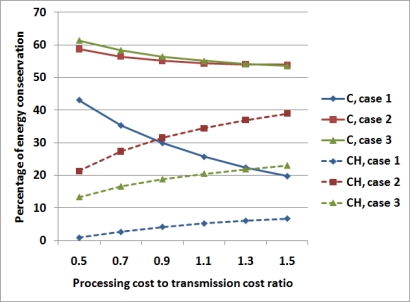
Percentage of energy conservation with respect to the ratio between processing cost and the cost to transmit 137 bytes of data, for cases UC-UE, NUC-UE and UC-NUE, for a density of 0.3 nodes per *m*^2^. Solid lines show energy conservation with respect to data processed only by the Coordinator; dashed lines show energy conservation with respect to data processed by every Cluster Head.

This fact is demonstrated by the results shown in Figure 7, which depicts the percentage of energy conservation while the ratio between the processing cost and the cost to transmit 137 bytes of data increases. The distance considered is equal to the average distance of all the nodes from the Coordinator. Comparison has been made both in the case that data are processed only by the Coordinator (solid lines) and cases in which data are processed by every Cluster Head (dashed lines). In the former, energy conservation decreases when processing cost to transmission cost ratio increases. In fact, as could be expected, when the processing cost increases with respect to the transmission cost, it becomes more convenient to transmit data rather than process data. On the other hand, when compared with the CH approach, energy conservation increases when the processing cost to transmission cost ratio increases. In fact, when the processing cost increases, it is more convenient to accurately choose the nodes where processing might be performed rather than processing data every time that it is possible to do so. Table 4 shows the results for OpA and OpB, for different node densities of 0.2 and 0.4 nodes/m^2^. The resulting tendency of an improved energy conservation when node density increases is basically due to two factors:

- in cases NUC-CE and UC-NUE, when the number of nodes in the same area increases, it is more likely that among neighbouring nodes there are node where the processing cost is lower;
- the higher the number of nodes in the same area, the higher the number of clusters formed, and therefore the bigger the amount of data that can be processed before they arrive to the Coordinator, reducing the energy cost.

It may be inferred from the results that using the framework would be particularly energy conserving when data from different nodes have to be processed together, the processing is pretty complex, and the energy consumption or the initial energy is not uniform for the network.

In the considered framework we have not addressed the routing problem and we have assumed that the routing of the packets is working correctly. However, computation of the paths and re-computation in case of failure is for sure another cause of energy consumption. When the network experiences the failure of a node, that node must be bypassed and data addressed to it must be sent to the following node. To do it, an appropriate new routing path must be found. The number of packets exchanged to find a new routing path depends on the routing algorithm used by the network. If we consider a bad scenario where an average of 50 packets have to be sent among 10-hop distant nodes where each node is 2 m far from the other ones, in case of one node failure every hour, energy conservation would decrease by about 7.8%. It has been estimated that the decrease in energy conservation for OpA and for a density of 0.3 nodes/m^2^ would be around 8.2% in case UC-UE, 12% in csae NUC-UE and 14.7% in case UC-NUE.

**Figure 8.**
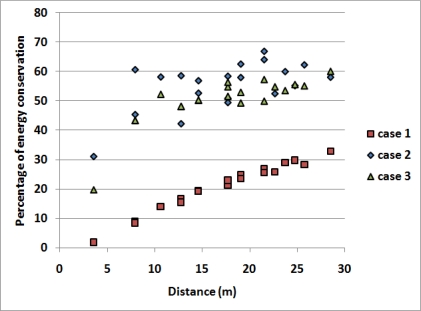
Percentage of energy conservation for every area of the network for OpA, comparison C, in cases UC-UE, NUC-UE and UC-NUE, for a density of 0.3 *nodes/m*^2^, for an increasing distance of the area from the Coordinator.

A final observation may be made in regard to Figure 8, which depicts the percentage of energy conservation for OpA, comparison C, in cases UC-UE, NUC-UE and UC-NUE, for a density of 0.3 nodes/m^2^, in relation to the distance of each area from the Coordinator. As could be expected, the greater the distance of the sources from the Coordinator, the more energy conservation is derived from the use of the framework.

Although they have not been reported, similar results have been obtained for all other cases.

## 6. Conclusions

In this paper we have studied the deployment of distributed applications inWireless Sensor Networks and proposed a solution aimed at minimizing the impact of energy consumption on the network lifetime. We have considered a scenario where the nodes have dissimilar capacities in terms of sensing and processing. The resulting algorithm has been implemented to perform simulations in different scenarios and the results have been compared with alternative solutions. We observed significant improvements in terms of energy savings. We may therefore infer that using the framework described would be particularly energy conserving when the application encompasses the processing of data coming from different nodes, the processing is pretty complex, and the energy consumption of nodes as well as battery energy is not uniform over the entire network.

## Figures and Tables

**Table 1.** Sub-operations for the *spatial and temporal monitoring example.*

**c*_i_***	**Description**
*c*_1_	Temperature sensing in area 1
*c*_2_	Temperature sensing in area 2
*c*_3_	Temperature sensing in area 3
*c*_4_	Temporal mean
*c*_5_	Spatial mean
*c*_6_	Only transmission
*c*_7_	No actions

**Table 2.** Sub-operations for OpA and OpB, for two monitored areas.

**c***_i_*	**OpA**	**OpB**
*c*_1_	Temperature, humidity, PH and light exposure sensing for area 1	
*c*_2_	Temperature, humidity, PH and light exposure sensing for area 2	
*c*_3_	Temporal mean	Aggregation of samples
*c*_4_	Spatial mean	Only transmissions
*c*_5_	Only transmission	No actions
*c*_6_	No actions	——

**Table 3.** Simulation setup parameters.

**Parameter**	**Value**
RF frequency	2,400 MHz
Bit rate	250 kbps
Programmable output power range	Programmable in 8 steps from approximately *−*24 to 0 dBm
Receiver sensitivity	*−*94 dBm
*P_R_*_0_	59.2 mW
*P_T_*_0_	26.5 mW
*η*	50%
*A*	14 dB
*e^instr^*	1 nJ
Packets header	12 bytes
Packets maximum payload	125 bytes

**Table 4.** Percentage values of energy conservation using the framework, for OpA and OpB, in cases UC-UE, NUC-UE and UC-NUE, comparisons C, CH and M, for a node density of 0.2 and 0.4 nodes/m^2^.

	**Node density [nodes/m^2^]**	**Case 1**[%]			**Case 2**[%]			**Case 3**[%]		
		**C**	**CH**	**M**	**C**	**CH**	**M**	**C**	**CH**	**M**
**OpA**	0.2 0.4	19.5 25.6	5.5 5.9	11 7 16 5	50.0 58.0	29.3 35.3	42.2 46.2	47.5 56.7	16.4 23.5	33.6 43.7
**OpB**	0.2 0.4	28.9 30.8	0.1 0.1	17.0 18.2	33.3 37.8	0.7 1.0	19.9 21.4	38.6 40.9	1.6 3.5	19.7 21.1

## Energy Consumption

*P_T_*: Transmitting radio frequency power consumption
*P_R_*: Receiving radio frequency power consumption
*P_T_*_0_: Transmitting circuit power consumption
*P_R_*_0_: Receiving circuit power consumption
*P_A_*: Power Amplifier power consumption
*P_Tx_*: Output power at the antenna
*P_Rx_*: Input power at the antenna
*δ*: Distance between transmitting and receiving nodes
*η*: Drain efficiency of the Power Amplifier
*A*: Parameter determined by the characteristics of the antennas such as gain and efficiency
*α*: Path loss exponent
*ε*: Parameter proportional to *P_Rx_* and *A*
*e**^tx^*: Transmission energy consumption
*R*: Data rate
*e_T_*: Energy consumed to send one bit
*e_R_*: Energy consumed to receive one bit
*e**^sens^*: Sensing energy consumption
*e**^proc^*: Processing energy consumption
*e**^instr^*: Average energy consumption per instruction executed
*M**^task^*: Number of instruction to process *task*
*smp**^in^*: Number of samples to be processed

**Problem Formulation**
*X* = {*x*_1_, ..., *x_i_*, ..., *x_N_*}: Set of *N* nodes in the WSN
**A** = (*a_ij_*) ∈ ℝ*^N^*^×^*^N^*: Adjacency matrix
**Δ** = (*δ_ij_*) ∈ ℝ*^N^*^×^*^N^*: Distances matrix
**E** = (*ε_ij_*) ∈ ℝ*^N^*^×^*^N^*: Matrix of the parameters *ε*
*V_i_* = {*P_R_*_0_*_i_*, *P_T_*_0_*_i_*, *η_i_*}: Set of characteristic parameters of the node *x_i_*
*F* = {*f*_0_, ..., *f_w_*, ..., *f_W_* }: Set of *W* tasks that can be performed by any node in the network
*D_i_* = {*d_i_*_1_, ..., *d_im_*, ..., *d_il_i__*}: Set of *l_i_* tasks that the node *x_i_* is able to perform
*O*: Operation performed by the WSN
*E**^tot^*: Total cost value associated to *O*
*C* = {*c*_1_, ..., *c_l_*, ..., *c_L_*}: Set of *L* sub-operations which must be executed by the nodes to perform operation *O*
*S* = {*s*_1_, ..., *s_i_*, ..., *s_N_*}: Set of *N* statuses, where *s_i_* corresponds to the status of the node *x_i_*

**Traffic Flows**
$T_{i}^{\mathrm{out}}=\left(t_{\mathrm{ih}}^{\mathrm{out}}\right)\in {𝕉}^{H}$: Vector of the output traffic flows generated by node *x_i_*
$t_{\mathrm{ih}}^{\mathrm{out}}=\left\{k_{\mathrm{ih}}^{\mathrm{out}},f_{\mathrm{ih}}^{\mathrm{out}}\right\}$: Traffic flow, corresponding to the *h*-th element of $T_{i}^{\mathrm{out}}$, where each sample of $k_{\mathrm{ih}}^{\mathrm{out}}$ bits is transmitted at the frequency $f_{\mathrm{ih}}^{\mathrm{out}}$
$T_{i}^{\mathrm{in}}$: Vector of the input traffic flows, corresponding to the union of the traffic flows received by node *x_i_*
$p(T_{i}^{\mathrm{in}},s_{i})$: Function which generates the output traffic $T_{i}^{\mathrm{out}}$, based on the input traffic and the status of the node *x_i_*

**Virtual Nodes**
*G_i_*: Set of sub-operations that a single node *x_i_* can perform consecutively for the implementation of the operation *O*
*L*: Set of sub-operations that the nodes cannot execute with the others
$X^{\mathrm{vir}}=\{x_{1}^{\mathrm{vir}},\ldots ,x_{v}^{\mathrm{vir}},\ldots ,x_{N^{\mathrm{vir}}}^{\mathrm{vir}}$: Set of *N**^vir^* nodes of the WSN with virtual nodes
**A***^vir^* ∈ ℝ*^N^vir^^*^×^*^N^vir^^*: Adjacency matrix for the WSN with virtual nodes
**Δ***^vir^* ∈ ℝ*^N^vir^^*^×^*^N^vir^^*: Distances matrix for the WSN with virtual nodes
**E***^vir^* ∈ ℝ*^N^vir^^*^×^*^N^vir^^*: Matrix of the parameters *ε* for the WSN with virtual nodes
$V_{v}^{\mathrm{vir}}$: Set of characteristic parameters of the node $x_{v}^{\mathrm{vir}}$ of the WSN with virtual nodes
$D_{v}^{\mathrm{vir}}$: Set of tasks that the node $x_{v}^{\mathrm{vir}}$ of the WSN with virtual nodes is able to perform

**Cost Functions**
$E_{i}^{\mathrm{sens}}$: Sensing cost function for node *x_i_*
γ*_i_*: Coefficient in inverse proportion with the residual energy of the node *x_i_*
$E_{i}^{\mathrm{proc}}$: Processing cost function for the node *x_i_*
$E_{i}^{\mathrm{tx}}$: Transmission cost function for the node *x_i_*
$Q_{l}^{\text{min}}$: Minimum number of nodes that have to perform the sub-operation *c_l_*
$Q_{l}^{\max}$: Maximum number of nodes that have to perform the sub-operation *c_l_*
